# The association between CHA_2_DS_2_-VASc score and erectile dysfunction: a cross-sectional study

**DOI:** 10.1590/S1677-5538.IBJU.2019.0058

**Published:** 2019-12-17

**Authors:** Dilay Karabulut, Umut Karabulut, Fatma Nihan Çağlar, Mithat Ekşi, Mustafa Gürkan Yenice, Ekrem Güner, Esra Dönmez Íşler, Ersan Oflar, Ali İhsan Taşçl, Faruk Aktürk

**Affiliations:** 1 Department of Cardiology, Istanbul BakIrkoy Dr. Sadi Konuk Educational and Training Hospital, Istanbul, Turkey; 2 Department of Cardiology, Istanbul, Acibadem International Hospital, Istanbul, Turkey; 3 Department of Urology, Istanbul Bakirkoy Dr. Sadi Konuk Educational and Training Hospital, Istanbul, Turkey

**Keywords:** Erectile Dysfunction, Vasectomy, Vas Deferens

## Abstract

**Purpose:**

This study aims to assess the association between CHA_2_DS_2_-VASc score and erectile dysfunction in patients who were admitted to cardiology outpatient clinics.

**Materials and methods:**

One hundred and two male patients who were admitted to the cardiology outpatient clinic were included to the study. Erectile dysfunction was evaluated in the urology outpatient clinic in the same hospital and scored using Turkish Version of The International Index of Erectile Function. CHA_2_DS_2_-VASc score was calculated for every patient using the current associated guidelines.

**Results:**

There was a negative correlation between The International Index of Erectile Function score and CHA_2_DS_2_-VASc score, age, hypertension, heart failure, diabetes mellitus, stroke respectively. Smoking and dislipidemia were not correlated with The International Index of Erectile Function score (p>0.05).

**Conclusion:**

CHA_2_DS_2_-VASc score can be used to detect Erectile dysfunction in patients who are admitted to the cardiology outpatient clinics.

## INTRODUCTION

Erectile dysfunction (ED) is defined as the inability to achieve or maintain an erection long enough to engage in sexual intercourse, affecting approximately 18% to 40% of men, aged 20 years and older (1, 2). In many cases, a single cause of ED is not easy to determine, and ED is more of a result of several overlapping conditions, such as anatomycal pathologies, genetics, systemic disorders, lifestyle, and environment (3).

ED and cardiovascular disease (CVD) are considered two manifestations of the same systemic disorder because they share similar risk factors and pathophysiologies. Nevertheless, in a significant number of patients with CVD, ED is also observed, which is believed to be associated with alterations of vascular system, endothelial dysfunction, inflammation, oxidative and emotional stress or sympathetic activation (4–7). Another factor strongly associated with ED is the limitation of the blood flow in penile arteries caused either by obstruction associated with atherosclerosis or by microthrombi (3).

CHADS2 and CHA_2_DS_2_-VASc scores are simple risk assessment schemes for thromboembolic stroke risk. CHADS2 was developed by investigators of stroke prevention in atrial fibrillation (SPAF) trial and includes stroke risk factors like, recent heart failure (HF), hypertension (HT), age >75 years, Diabetes Mellitus (DM) and history of stroke or transischemic attack (TIA) (8). These two risk scores are most widely used to assess the severity of thrombotic changes and the risk of thrombosis in atrial fibrillation (AF) patients (9, 10). Both scores are not only predictive of thromboembolic stroke risk, but also associated with adverse vascular function, CVD risk, and total death in the whole AF population (11–13). Although the prediction of ED is not the primary aim of the CHA_2_DS_2_-VASc score, its components such as older age, DM, HT, HF, and prior vascular disease are all important prognostic factors for endothelial dysfunction. Based on this knowledge, in this study, we aimed to assess the relationship between CHA_2_DS_2_-VASc score and ED in patients who were admitted to cardiology outpatient clinics.

## MATERIALS AND METHODS

One hundred-two male patients, older than 18 years-old, who were admitted to the BakIrköy Dr. Sadi Konuk Research and Training Hospital cardiology outpatient clinic were included into the study. Patient's demographic and clinical characteristics were recorded ED of patient was evaluated in urology outpatient clinic in the same hospital.

Patients were excluded from the study if they had: a history of prostatectomy or other procedures associated with a loss of sexual function, current use of phosphodiesterase type 5 (PDE-5) inhibitors, and beta blockers.

ED was evaluated using by Turkish Version of The International Index of Erectile Function (IIEF) with 5 questions (14). It is a self-administered questionnaire which patient's responses are based on their experience during the last 4 weeks, and is scored on a 5-point Likert scale; lower values represent poorer sexual function. Total scores are classified as: 22-25: No ED, 17-21: Mild ED 12-16: Mild to moderate ED 8-11: Moderate ED 5-7: Severe ED (15). This questionnaire provides data on ED with 98% sensitivity and 88% specificity (16).

CHA_2_DS_2_-VASc scores were calculated for every patient based on predefined point systems according to the current guidelines for scoring and diagnosing all mentioned conditions. In the CHA_2_DS_2_-VASc score, 2 points were assigned if a patient had a history of stroke or TIA or was ≥ 75 years of age. One point was assigned for the age 65-74 years, female sex, history of HT, DM, recent HF, and vascular disease (history of myocardial infarction, presence of complex aortic plaque, or peripheral artery disease) (8, 17).

According to their CHA_2_DS_2_-VASc score, the patients were divided into two groups: group 1: 0-3 points; group 2: ≥ 3.

In accordance with the Declaration of Helsinki, the study protocol was approved by the Regional Ethics Committee. Before the study entry, a written informed consent was obtained from every study participant.

### Statistical analysis

SPSS 22.0 (IBM Corporation, Armonk, New York, United States) program was used for variable analyses. All data were analyzed for normality of distribution using the Kolmogorov--Smirnov test. Continuous variables were summarized as mean±standard deviation (SD). Pearson and Spearman correlation test was used to evaluate the correlation between the parameters. P value <0.05 was considered statistically significant.

## RESULTS

The clinical and demographic data of the patients are summarized in Table-1. There was a negative correlation between IIEF score and CHA-_2_DS_2_-VASc score, age, HT, HF, DM, stroke respectively (r=-0.652, p=0.00, r=-0.484 p=0.00 r=-0.414, p=0.00, r=-0.278, p=0.007, r=-0.241, p=0.019, r=- 241 p=0.018). However, smoking and dislipidemia were not correlated with patients’ IIEF score (p >0.05) (Table-2).

**Table 1:** The clinical and demographic data.

	Minimum	Maximum	Mean	Std.
Age (year)	28	76	52.8	10.7
Height (cm)	158	185	171.6	5.4
Weight (kg)	55	170	80.7	13.8
BMI (kg/m^2^)	21	55.5	27.3	4.4
IIEF	5	25	15.1	5.1
CHA2DS2-VASc	1	6	2.1	1.1

**STD** = Standart deviation; **BMI** = Body Mass Index; **IIEF:** International Index of Erectile Function

**Table 2:** Correlation between IIEF score and clinical parameters.

IIEF		
**Hypertension**	Pearson Correlation	-0.414**
	Sig. (2-tailed)	0.000
**Congestive Heart Failure**	Pearson Correlation	-0.278**
	Sig. (2-tailed)	0.007
**Diabetes Mellitus**	Pearson Correlation	-0.241*
	Sig. (2-tailed)	0.019
**Stroke**	Pearson Correlation	-0.241*
	Sig. (2-tailed)	0.018
**Age**	Pearson Correlation	-0.484**
	Sig. (2-tailed)	-0.000

* means p < 0.005;

** means p < 0.001;

** IIEF: International Index of Erectile Function

When patients were divided into 2 groups according to the CHA_2_DS_2_-VASc scores, the mean IIEF score was 17.76±4, 29 in group 1 (n=55) and 11.98±4, 21 in group 2 (n=47) respectively. In the group of patients with CHA_2_DS_2_-VASc score 3 and above IIEF scores were found significantly lower (p=0.014).

## DISCUSSION

In this study we found a inverse correlation between IIEF score and CHA2DS-2VASc, age, HT, CHF, DM, stroke respectively and smoking and dislipidemia were not correlated with patient's IIEF scores.

The CHA_2_DS_2_-VASc score was initially developed for stroke risk stratification in patients with AF. However, it has recently been applied to different clinical conditions. For example, it has been used to estimate the risk of stroke and the development of thromboembolism inpatients with heart failure who are in sinus rhythm (18). It has also been used to estimate the risk of stroke following by-pass surgery and stroke without AF in the general population (18–20). Although the prediction of erectile dysfuction is not the primary aim of the CHA_2_DS_2_-VASc score, its components such as older age, DM, HF, and prior vascular disease are all important prognostic factors for endotetial dysfunction.

The association between ED and CVD, particularly coronary artery disease (CAD), has been demonstrated in previous studies (21, 22). In a meta-analysis of a prospective cohort study, ED was significantly and independently associated with an increased risk of 48% for CVD, 46% for CAD, 35% for stroke, and 19% for all-cause mortality (21). Montorsi et al. proposed that atherosclerosis affects all major vascular beds equally, including penile and coronary arteries (23). Another explanation for the association between ED and CVD is inflammation and endothelial dysfunction (24–26). Vlachopoulos et al. demonstrated that low-grade subclinical inflammation affected endothelial function and led to a prothrombotic including von Willebrand factor, tissue plasminogen activator, and fibrinogen in individuals with or without CVD (26). The relationship between ED and CVD is complex, and ED and CVD should be considered two clinical manifestations of the same systemic disorder (27).

On the other hand, in this study, it has been shown that the frequency of ED is increased in patients with high risk of CVD. We think that male patients who are admitted to the cardiology clinics do not express their complaints about ED. Therefore, it may be important to refer patients with high CHA_2_DS_2_-VASc score to urology outpatient clinics for early diagnosis of ED.

The cross-sectional design of the study and the number of patients are considered to be important limitations of this study, however to our knowledge this is the first study which evaluates the ED with CHA_2_DS_2_-VASc score in patients without AF.

In conclusion, we postulated that CHA- _2_DS_2_-VASc score can be used to detect the ED in patients who are admitted to the cardiology departments.
